# Serum Bilirubin Concentration in Healthy Adult North-Europeans Is Strictly Controlled by the UGT1A1 TA-Repeat Variants

**DOI:** 10.1371/journal.pone.0090248

**Published:** 2014-02-28

**Authors:** Marianne K. Kringen, Armin P. Piehler, Runa M. Grimholt, Mimi S. Opdal, Kari Bente F. Haug, Petter Urdal

**Affiliations:** 1 Department of Pharmacology, Oslo University Hospital, Ullevål, Oslo, Norway; 2 Fürst Medical Laboratory, Oslo, Norway; 3 Department of Medical Biochemistry, Oslo University Hospital, Ullevål, Oslo, Norway; 4 Institute of Clinical Medicine, University of Oslo, Oslo, Norway; Gentofte University Hospital, Denmark

## Abstract

The major enzyme responsible for the glucuronidation of bilirubin is the uridine 5′-diphosphoglucose glucuronosyltransferase A1 (UGT1A1) enzyme, and genetic variation in the UGT1A1 gene is reported to influence the bilirubin concentration in the blood. In this study, we have investigated which gene-/haplotype variants may be useful for genetic testing of Gilbert's syndrome. Two groups of samples based on serum bilirubin concentrations were obtained from the Nordic Reference Interval Project Bio-bank and Database (NOBIDA): the 150 individuals with the highest bilirubin (>17.5 µmol/L) and the 150 individuals with normal bilirubin concentrations (<17.5 µmol/L). The individuals were examined for the TA_6_>TA_7_ variant in the UGT1A1 promoter and 7 tag-SNPs in an extended promoter region of UGT1A1 (haplotype analysis) and in selected SNPs in candidate genes (SLCO1B3, ABCC2 and NUP153). We found significant odds ratios for high bilirubin level for all the selected UGT1A1 variants. However, in stepwise multivariate logistic regression analysis of all genetic variants together with age, sex, country of origin and fasting time, the repeat variants of UGT1A1 TA_6_>TA_7_ and SLCO1B3 rs2117032 T>C were the only variants significantly associated with higher bilirubin concentrations. Most individuals with high bilirubin levels were homozygous for the TA_7_-repeat (74%) while only 3% were homozygous for the TA_7_-repeat in individuals with normal bilirubin levels. Among individuals heterozygous for the TA_7_-repeat, a low frequent UGT1A1-diplotype harboring the rs7564935 G-variant was associated with higher bilirubin levels. In conclusion, our results demonstrate that in testing for Gilbert's syndrome, analyzing for the homozygous TA_7_/TA_7_-genotype would be appropriate.

## Introduction

High levels of bilirubin in serum or plasma may indicate increased degradation of hemoglobin (hemolytic disease), reduced transport of bilirubin into the liver, reduced glucoronidation of bilirubin or be a sign of hepato-biliary disease. The major enzyme responsible for the glucuronidation of bilirubin is the uridine 5′-diphosphoglucose glucuronosyltransferase A1 (UGT1A1) enzyme and genetic variation in the UGT1A1 gene is reported to influence the serum/plasma bilirubin concentration [1]. The most common variant in UGT1A1 is a TA -insertion (TA_6_>TA_7_) in the promoter of UGT1A1, which is seen in individuals with low grade hyperbilirubinemias (Gilbert's syndrome, also called Gilbert-Meulengracht syndrome) [1]–[4]. In individuals with moderately increased serum bilirubin concentration it is important to determine whether this increase is due to genetic variations in UGT1A1 or other genes, or is caused by liver disease. Use of genetic testing for Gilbert's syndrome has been suggested [5]. This requires sufficient knowledge on which gene variants to test for as well as data on prevalence and penetrance of the gene variants included in the testing. At present, satisfactory data are only available for the prevalence of a few specific gene variants. Here, we present additional data and put forward a proposal on which genes and which gene variants to be included in diagnostic testing as well as the penetrance of the gene variants chosen. We are convinced this information will improve the use of genetic tests to detect or exclude Gilbert's syndrome.

## Results

The distribution of serum bilirubin concentrations in the total NORIP population is skewed towards high values, and the 91.5 percentile of 17.5 µmol/L separates the 150 samples with high bilirubin from the 150 samples with normal bilirubin (Figure S1). The 17.5 µmol/L was therefore used as a cut-off to separate the two bilirubin groups. Compared to the normal bilirubin group, the individuals in the high bilirubin group were younger (mean age 45 and 53 years, respectively), contained more men (56 and 45%, respectively) had fasted for a longer time (mean 11 and 9 hours), and were mostly Finns.

The genotype of a repeat variant (rs8175347) and 7 single nucleotide polymorphisms (SNP's) in the UGT1A1 promoter (rs2003569, rs4124874, rs17862878, rs13009407, rs17862875, rs17862874, rs7564935), as well as SNPs within 3 other genes, the solute carrier organic anion transporter 1B3 (SLCO1B3) (rs2117032, rs17680137), the ATP-binding cassette transporter C2 (ABCC2) (rs717620) and the nucleoporin gene (NUP153) (rs2328136), were determined in all the 300 samples. The allele frequencies of these variants are shown in Table 1 together with published frequencies in North-Europeans. None of the SNPs in UGT1A1, SLCO1B3, ABCC2 or NUP153 deviated from the Hardy-Weinberg equilibrium.

**Table 1:** Frequencies and chromosomal localization of SNPs in normal and high bilirubin individuals.

	Chromosomal location GRCh37/hg19		Minor allele frequency				P-values	
Gene (SNP)	Chromosome	Nucleotide position	Normal Bilirubin	High Bilirubin	Bilirubin Total^a^	Caucasians	HWE P-value^a^	P-value^c^
UGT1A1								
Rs8175347 (TA_6_>TA_7_)	2	234668881	0.30	0.85	0.35	0.31–0.39^b^	0.20	0.01
Rs2003569 (G>A)	2	234667937	0.12	0.01	0.11	0.13–0.18	0.51	0.01
Rs4124874 (T>G)	2	234665659	0.43	0.87	0.47	0.39–0.56	0.95	0.01
Rs17862878 (G>A)	2	234661948	0.05	0.01	0.05	0.08	0.11	0.01
Rs13009407 (C>G)	2	234652347	0.23	0.64	0.27	0.23–0.26	0.20	0.01
Rs17862875 (G>A)	2	234649302	0.29	0.77	0.33	0.30–0.32	0.15	0.01
Rs17862874 (A>G)	2	234648746	0.07	0.01	0.07	0.05–0.06	0.43	0.01
Rs7564935 (G>T)	2	234645186	0.36	0.77	0.40	0.37	0.50	0.01
SLCO1B3								
Rs2117032 (T>C)	12	21074122	0.34	0.42	0.35	0.33–0.37	0.91	0.08
Rs17680137 (C>G)	12	21015906	0.14	0.16	0.14	0.17–0.19	0.43	0.51
ABCC2								
Rs717620 (C>T)	10	101542578	0.16	0.18	0.16	0.15–0.23	0.35	0.54
NUP153								
Rs2328136 (G>A)	6	17709551	0.21	0.19	0.21	0.16–0.20	0.27	0.55

Minor allele frequencies for Caucasians were obtained from dbSNP at http://www.ncbi.nlm.nih.gov/SNP or from references indicated.

a Bilirubin Total frequencies and HWE P-values were estimated based on the total NORIP population: The normal and high bilirubin groups represent the 91.5 and 8.5 percentiles of the total NORIP population, respectively.

b Ref:[24], [25].

c Pearson chi-square test was used to compare SNP frequencies between normal- and high bilirubin individuals. P-values were adjusted for multiple comparison by false discovery rate (FDR); q<0.05.

### UGT1A1 Variants

**Figure 1:**
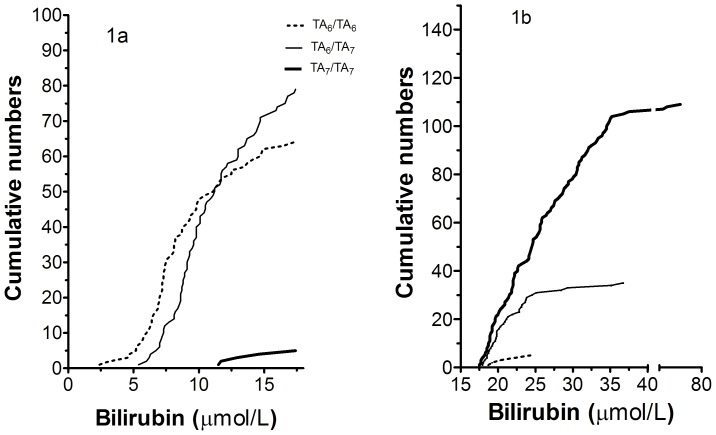
Cumulative numbers of UGT1A1 rs8175347 genotypes according to bilirubin concentration in individuals with A) normal bilirubin and B) high bilirubin (n = 150 in each group). The thick line represents the TA_7_/TA_7_ genotype, the thin line represents the TA_6_/TA_7_-genotype and the stippled line represents the TA_6_/TA_6_-genotype.

Among the well-known UGT1A1 TA-repeat variant, the TA_6_>TA_7_ allele (also named UGT1A1*28) was the only variant found in this study, while the rare TA_6_>TA_5_ (UGT1A1*36) or TA_6_>TA_8_ (UGT1A1*37) alleles were not found. In the normal bilirubin group, the TA_6_-containing genotypes (TA_6_/TA_7_ and TA_6_/TA_6_) were the dominant ones (79 and 64 out of 148, Figure 1A) and were the only genotypes found below 11.5 µmol/L (51 and 51, respectively). The TA_7_/TA_7_ genotype was found in 5 individuals (3%). The bilirubin in these 5 individuals were all above 11.5 µmol/L, representing the upper third of the bilirubin distribution. By contrast, in the high bilirubin group (Figure 1B) the TA_7_-genotypes (TA_7_/TA_7_ and TA_6_/TA_7_) clearly dominated (109 and 35 out of 148, respectively) and were the only genotypes above 24.3 µmol/L (62 and 6, respectively). Since the normal and high bilirubin groups represent 91.5 and 8.5% of the NORIP bilirubin distribution, it can be estimated that the TA_6_/TA_6_ -genotype occurs in 39.8%, TA_6_/TA_7_ in 50.8% and TA_7_/TA_7_ in 9.3% of the NORIP population. Likewise, the penetrance for high bilirubin was estimated to be 1% for the TA_6_/TA_6_ -genotype, 4% for TA_6_/TA_7_ and 67% for TA_7_/TA_7_ in the NORIP population. From the data of Figure 1, we have estimated the cumulative distribution of TA_6_/TA_6_, TA_6_/TA_7_ and TA_7_/TA7-carriers with increasing serum bilirubin concentration (See Table S1). Sub-grouped by country, the estimated frequencies of the TA_7_-allele were 33, 34, 34 and 40% in Norwegians, Danes, Finns and Swedes respectively. However, in the high bilirubin group, Finns harboring the TA_7_/TA_7_ –genotype were most frequent (45% compared to 15–21%).

**Figure 2:**
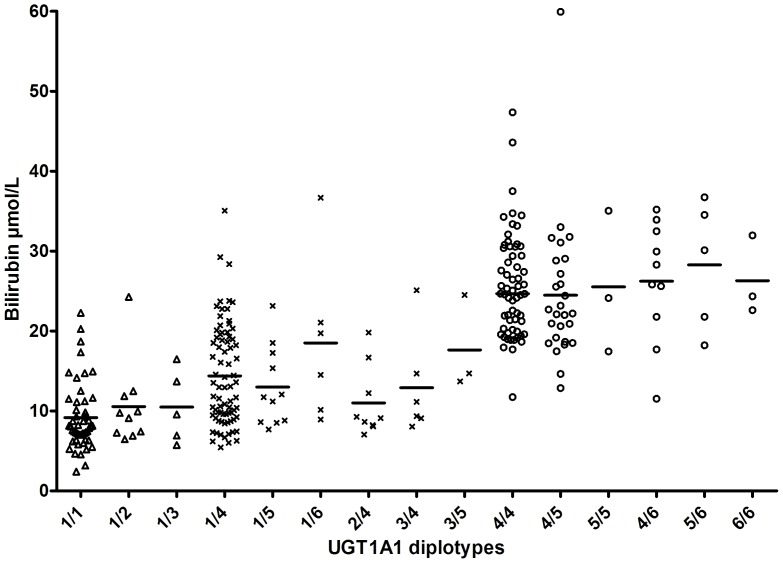
The Relationship between median bilirubin concentrations and UGT1A1 diplotypes. UGT1A1 rs8175347genotypes are marked in triangles (TA_6_/TA_6_), crosses (TA_6_/TA_7_) and circles (TA_7_/TA_7_).

Four of the seven UGT1A1 promoter tag-SNPs were in close linkage disequilibrium with the TA_6_>TA_7_-allele (r^2^>0.5). All the 8 UGT1A1 variants differed considerably in their allele frequencies between the normal and high bilirubin group (Table 1). From the total eight UGT1A1 promoter variants analyzed in this study, we identified nine haplotypes. The frequencies of the six haplotypes occurring more than once are shown in Table 2. The most common haplotypes were haplotypes #1 (estimated frequency of 0.52) and #4 (estimated frequency of 0.27), harboring the TA_6_ or TA_7_-alleles in the UGT1A1, respectively. Haplotype pairs (diplotypes) were assigned to each individual and plotted against corresponding bilirubin concentration (Figure 2). All diplotypes harboring the TA_7_-allele, except diplotype 2/4 (homozygous for the rs7564935 T-allele), had significantly higher bilirubin concentrations compared to diplotype 1/1 in the TA_6_/TA_6_-group. All diplotypes homozygous for TA_7_ exhibited higher bilirubin than any other diplotypes. An individual effect of UGT1A1 rs4124874 (c.-3279T>G) on bilirubin concentration was not found in our population, as seen in Figure 2 where diplotype 1/2 and 1/3 harbour the rs4124874 G-allele together with the rs8175347 TA_6_- allele.

**Table 2:** Haplotype structure of UGT1A1 tagSNPs and haplotype frequencies.

#	Rs7564935	Rs17862874	Rs17862875	Rs13009407	Rs17862878	Rs4124874	Rs2003569	Rs8175347	Normal Bilirubin Frequency (n)	High Bilirubin Frequency (n)	Bilirubin Total Frequency^a^
	−23695	−20135	−19579	−16534	−6933	−3222	−944	0			
1	G	A	G	C	G	T	G	(TA)_6_	0.59 (168)	0.14 (39)	0.52
2	T	G	G	C	G	G	A	(TA)_6_	0.06 (17)	0.01 (2)	0.06
3	G	A	G	C	A	G	A	(TA)_6_	0.04 (12)	0.01 (2)	0.04
4	T	A	A	G	G	G	G	(TA)_7_	0.24 (68)	0.64 (183)	0.27
5	T	A	A	C	G	G	G	(TA)_7_	0.05 (15)	0.13 (37)	0.06
6	G	A	G	C	G	G	G	(TA)_7_	0.01 (4)	0.08 (23)	0.02

The numbers below tagSNPs indicate the distance in bp from Rs8175347. Three haplotypes were identified only once and are therefore not shown in table.

a Bilirubin Total frequencies were estimated based on the total NORIP population: The normal and high bilirubin groups represent the 91.5 and 8.5 percentiles of the total NORIP population respectively.

### Other Gene Variants

Only one SNP in the SLCO1B3 gene (rs2117032 (T>C) tended to be more frequent in the high bilirubin group compared to the normal bilirubin group (P = 0.08, Table 1). Four haplotypes were identified for the two SLCO1B3 variants (data not shown), however, assigned diplotypes were similar with respect to bilirubin concentrations. The allele frequencies of SLCO1B3 rs17680137 (C>G), ABCC2 rs717620 (C>T) and NUP153 rs2328136 (G>A) were equally frequent in both bilirubin groups.

### Multivariate Analysis

**Figure 3:**
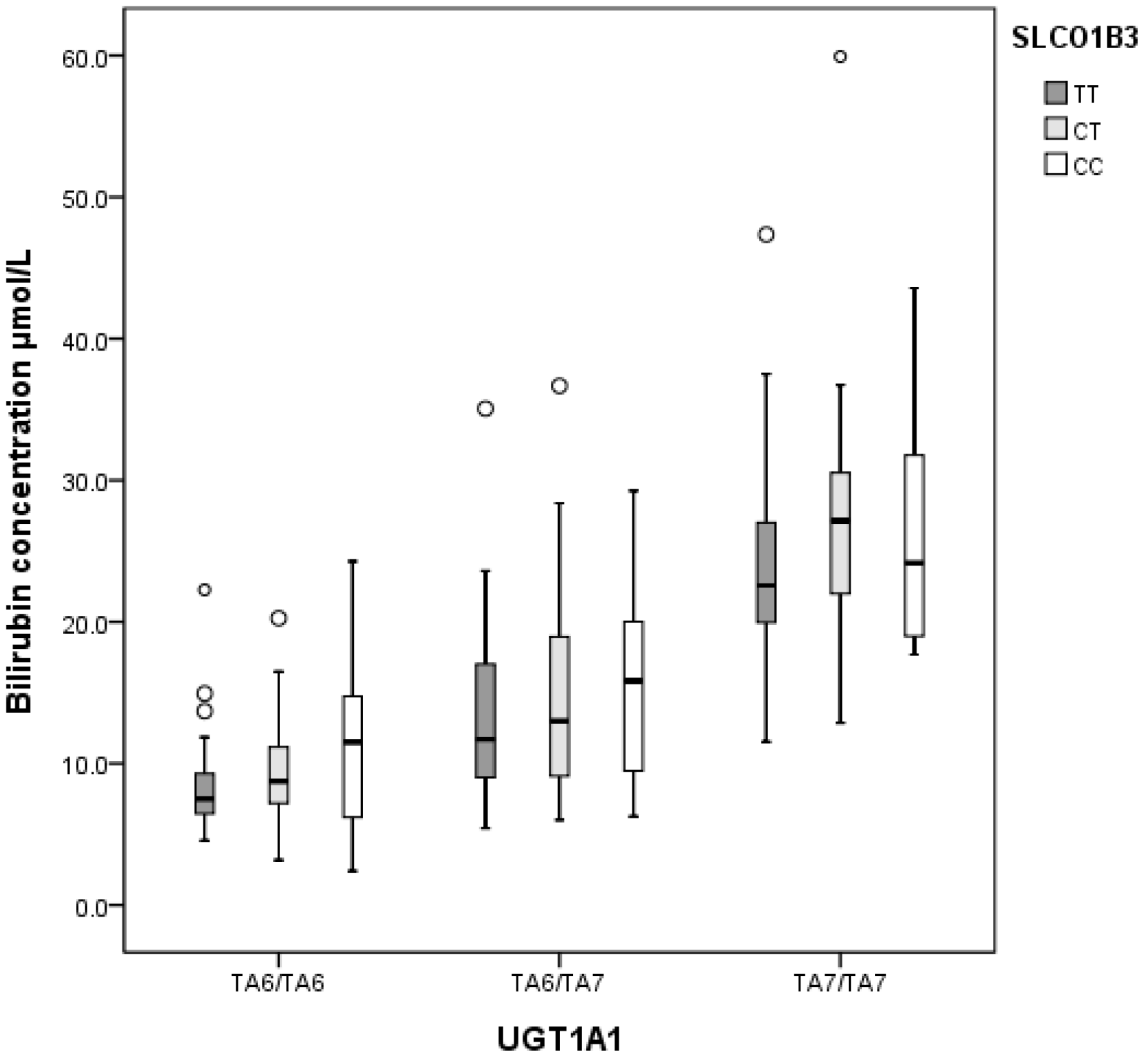
Box plot of bilirubin for different genotypes of UGT1A1 TA_6_/TA_7_ and SLCO1B3 T>C. The box represents the values from the 25 to 75% percentile (interquartile range; IQR). The middle line represents the median. The vertical line extends from the minimum to the maximum value, excluding outliers (>1.5 IQR of the 75% percentile) which are marked as open circles.

In stepwise multivariate logistic regression analysis of all genetic variants together with age, sex, country of origin and fasting time, the repeat variants of UGT1A1 TA_6_>TA_7_ and SLCO1B3 rs2117032 T>C were significantly associated with higher bilirubin concentrations (Table 3). Box-plot of bilirubin concentrations for the combinations of UGT1A1 TA_6_>TA_7_ and SLCO1B3 (rs2117032, T>C) genotypes, in the normal and high bilirubin groups combined, is shown in Figure 3. Of the Nordic countries, the Finns showed the highest odds for high bilirubin levels (Table 3). Furthermore, when individuals were stratified according to TA_6_- and TA_7_-genotype groups, Finns showed a higher frequency of individuals with the TA_6_/TA_7_-genotype in the high bilirubin group (47%) compared to the other countries (17–30%). On the other side, Norwegians had the highest frequency of individuals with the TA_7_/TA_7_-genotype in the low bilirubin group (11% compared to 0–3%).

**Table 3:** The Odds ratio (OR) for high bilirubin levels.

	Crude		Adjusted	
n = 289	OR (95% CI)	P-value	OR (95% CI)	P- value
Age (yr)	0.98 (0.97–0.99)	<0.0001	0.97 (0.95–0.99)	0.007
Sex				
Female	1.00		1.00	
Male	1.54 (0.97–2.42)	0.065	3.43 (1.48–7.92)	0.004
Fasting time (hr)	1.11 (1.05–1.18)	<0.0001	1.15 (1.03–1.27)	0.011
Country				
Norway	1.00		1.00	
Denmark	1.37 (0.67–2.81)	0.394	3.48 (0.88–13.82)	0.076
Sweden	1.75 (0.86–3.57)	0.123	3.32 (0.85–13.07)	0.086
Finland	3.32 (1.71–6.45)	<0.0001	5.82 (1.56–21.67)	0.009
UGT1A1 (Rs8175347)				
TA_6_/TA_6_	1.00		1.00	
TA_6_/TA_7_	7.09 (2.39–20.99)	<0.0001	11.88 (2.97–47.47)	<0.0001
TA_7_/TA_7_	348.8 (90.38–1346.18)	<0.0001	1253.75 (196.88–7983.99)	<0.0001
SLCO1B3 (Rs2117032)				
T/T	1.00		1.00	
C/T	1.34 (0.81–2.22)	0.25	1.35 (0.55–3.28)	0.51
C/C	2.09 (1.01–4.32)	0.047	5.75 (1.65–20.04)	0.006

- 2 Log likelihood: 165.36.

A multivariate logistic regression analysis was also made with the UGT1A1 TA_6_>TA_7_ variant exchanged with the various UGT1A1 diplotypes seen in Figure 2 (with 1/1 as the reference diplotype). Among individuals with the TA_6_/TA_7_-genotype, diplotype 1/6 showed a significantly higher odds ratio for high bilirubin levels than any of the other diplotypes (data not shown). Diplotype 1/6 is homozygous for the rs7564935 G-allele.

## Discussion

We confirmed the well-known close association between the TA_7_-repeat variant of the UGT1A1 promoter and high serum bilirubin concentrations [1]–[3]. In our population of healthy Nordic individuals, this variant allele was present only in the upper forty percent of the serum bilirubin concentration distribution and was the most dominant repeat variant when serum bilirubin was above 25–30 µmol/L.

The other UGT1A1 promoter gene variants (tag SNPs) studied covaried to a large extent with the UGT1A1 TA_6_>TA_7_ variant, but in multivariate logistic regression analysis their effect upon serum bilirubin concentration became non-significant. In previous studies, the UGT1A1 rs4124874 (c.-3279T>G) has been found to have an individual effect on bilirubin concentration [6], [7], however, in our study the presence of the rs4124874 G-allele alone was not associated with increased bilirubin concentration. Therefore, our results strongly indicate that the TA_7_-allele of rs8175347 is the predominant UGT1A1 variant being responsible for increased bilirubin concentrations in our population, and not rs4124874.

Among the four SNP's located in other genes, only the C/C-genotype of SLCO1B3 (rs2117032) was related to high serum bilirubin concentration, which is in agreement with recent literature [8]. The previous described association of rs17680137 (SLCO1B3), with increased bilirubin concentrations [8], was not confirmed in our study. The two SNPs in SLCO1B3 are probably of less significance in the case of genetic testing for Gilbert's syndrome, as a large majority of the TA_6_/TA_6_-carriers and TA_6_/TA_7_-carriers with these SNPs showed bilirubin concentration well below 25 µmol/L.

We did not find any association between bilirubin levels and ABCC2 (rs717620) or NUP-153 (rs2328136). ABCC2 mediates transport of a wide range of anionic substrates into the bile, including conjugated bilirubin [9], and an intronic polymorphism (rs717620) has been associated with reduced ABCC2 promoter activity [10]. However, we found no correlation between this SNP and bilirubin concentration in our samples. Furthermore, we could not confirm a correlation between *NUP-153* (rs2328136) and lower bilirubin concentration which was found in an Indian population [11]. We found a small increase, rather than decrease, in bilirubin concentration in our study (data not shown).

The prevalence of Gilbert's syndrome has repeatedly been reported to be in the range of 6–8% [12], [13], and our estimate of the TA_7_/TA_7_–genotype being found in 9.3% of the Nordic population as a whole is in agreement with this statement. However, the prevalence of the TA_7_/TA_7_-genotype varies much between the Nordic countries, being highest in Finland and lowest in Norway. Interestingly, the country-specific serum bilirubin upper reference limit reported in the NORIP study varied similarly, being 30 µmol/L in Finland and 25, 24 and 21 µmol/L in Sweden, Denmark and Norway, respectively. The overall upper reference limit was calculated to 25 µmol/L [14]. Given the close relationship between TA_7_/TA_7_ and high serum bilirubin concentrations, the finding that Finns having high mean bilirubin concentration also had the highest prevalence of the TA_7_/TA_7_-genotype was not unexpected. In the multivariate logistic regression modelling (Table 3), a model without adjustment for country influenced the OR for high bilirubin of the TA_7_/TA_7_-genotype more than 40%, indicating that the high prevalence of the TA_7_/TA_7_-genotype in Finns could be a confounding factor. Additionally, the finding that Finns had a significantly higher bilirubin concentration in individuals with the TA_6_/TA_7_-genotype compared to Norwegians, indicate that other factors, not measured in this study, are more prevalent in Finns.

When gene testing for Gilbert's syndrome, we will from time to time identify the TA_6_/TA_7_-genotype. What is the significance of such a finding? Clearly, the TA_6_/TA_7_-genotype in general shows higher serum bilirubin levels than does TA_6_/TA_6_. But nevertheless, the data in Figure 2 does not suggest one specific genetic constellation added to TA_6_/TA_7_ that gives rise to very high bilirubin concentrations. By not including the TA_6_/TA_7_-genotype as an explanation to Gilbert's syndrome, there will be a definite, but small, rate of false negative results. Alternatively, by including TA_6_/TA_7_ there is an obvious risk of many false positive results. A false positive result appears less acceptable than a false negative result, since a false positive result wrongly suggests Gilbert's syndrome and might stop the diagnostic process, whereas a false negative result will only lead to additional, but probably in the end, unnecessary examinations.

Knowledge about prevalence and penetrance of the gene variants responsible for Gilbert's syndrome is a prerequisite for useful genetic testing [5]. We suggest that the UGT1A1*28-variants are the only gene variants to test for. The prevalence of this variant is well known. The penetrance, i.e. the fraction of the TA_7_/TA_7_-genotype above a defined bilirubin decision value, is now possible to estimate from our distribution data of TA_6_/TA_6_-, TA_6_/TA_7_- and TA_7_/TA_7_-genotypes with increasing serum bilirubin concentration. As for the TA_7_/TA_7_-genotype these data should be particularly precise above 17.5 µmol/L, which is the concentration range where genetic testing for Gilbert's syndrome might be requested.

In addition to bilirubin, UGT1A1 also combines with several medical drugs such as statins, opioids, steroids, thyroid hormones and anticancer drugs to form the corresponding glucuronides as phase II reactions of drug metabolism [4]. The glucuronides are mostly less biologically active and more water-soluble than the parent compound. Genetic variants in UGT1A1 can thus possibly alter the metabolism of these drugs leading to accumulation of the drug in the body causing both increased side effects and possible toxicity. This has been shown for the active metabolite SN-38 of the anticancer drug irinotecan. Patients who are homozygous for the UGT1A1 TA_7_-allele have an increased risk for inrinotecan induced toxicity like neutropenia [15]. Because of this, The US Food and Drug Administration (FDA) has recommend reduced starting dose for patients with this genotype. However, it remains to be tested if the different haplotypes found in our study will give rise to increased toxicity of SN-38.

## Materials and Methods

### Population

The Nordic reference interval project (NORIP), organized by the Nordic Society of Clinical Chemistry and involving 102 laboratories from Denmark, Finland, Iceland, Norway and Sweden, collected blood samples from 3035 healthy individuals during the years 2000–2001 [16]. Each of the laboratories included 25–50 persons, evenly distributed in gender and four age groups (18–30, 31–50, 51–70 and above 70 years). In serum and plasma, bilirubin and 24 other of the most common quantities used in medical biochemistry were measured, and reference intervals for these quantities were calculated [14], [16]. During this process, 28 individuals were excluded according to predefined exclusion criteria; in most cases, because two or more of an individual's 25 serum components were considered outliers based on statistical considerations. All laboratories were invited to send their blood samples for establishment of the Nordic Reference Interval Project Bio-bank and Database (NOBIDA). Together, 94 laboratories sent their samples (buffy coat from 5 mL of EDTA-blood, serum and plasma) from 2500 individuals. Located in Denmark and stored below −60°C, NOBIDA samples can be applied for to correlate reference values to genotypes [17]. At the time of our study, buffy coat samples from 1800 individuals were available. We considered these 1800 individuals as representative for the NORIP population since the samples from the 700 missing individuals had been removed by random sampling for use in various reference interval studies. For the present study, we obtained two groups of samples from NOBIDA based on serum bilirubin concentrations: (i) buffy coat from the 150 individuals with the highest bilirubin (>17.5 µmol/L) and (ii) 150 random samples from the remaining 1650 NOBIDA samples (<17.5 µmol/L). We also received all NORIP-data available of these 300 samples/individuals.

### Ethics Statement

Written informed consent for use with NORIP and NOBIDA was obtained from all subjects before blood samples were drawn. The study was approved by Regional Ethics Committee in all five countries. In addition the Norwegian Regional Ethics Committee approved this retrospective study. Patient records/information was de-identified prior to analysis.

### Gene Variant Selection

#### UGT1A1

The TA-repeat variant in the promoter of UGT1A1 (rs8175347) has a well-documented effect on serum bilirubin concentration [2] and was therefore included in this study. Furthermore, Single Nucleotide Polymorphisms (SNPs) located in the upstream region of UGT1A1 exon 1, but downstream of UGT1A3 exon 1 (∼30 Kbp), were selected using the Tagger program [18] from the HAPMAP website (http://www.hapmap.org) with the CEU dataset (release 24, phase II, Nov08). Based on the chosen criteria (pairwise tagging algorithm, r^2^≥0.9 and minor allele frequency of at least 0.05), seven tag-SNPs (rs2003569, rs4124874, rs17862878, rs13009407, rs17862875, rs17862874, rs7564935) were selected for genotyping.

#### Other Genes

Based on a non-systematic review of the literature concerning genetic variants affecting bilirubin levels in serum, several SNPs in different genes were selected: Rs2117032 and rs17680137 [8] located in SLCO1B3 that is involved in transport of unconjugated bilirubin into the liver, rs717620 [10] located in ABCC2 that is involved in transport of conjugated bilirubin out of the liver, and rs2328136 [11], recently found to be associated with bilirubin concentration variation and located in the gene coding for the nuclear protein NUP53.

### DNA Isolation

DNA was extracted from 100 µl buffy coat diluted with equal amount phosphate-buffered saline (PBS) using the MagNA Pure LC DNA Isolation Kit I (cat# 03 003 990 001, Roche, Basel, Switzerland) on a MagNA Pure LC Instrument (Roche). The DNA yield and purity were measured using a Nanodrop 1000 (Thermo Scientific, Waltham, USA). The average DNA concentration was 55.5 ng/µl and all samples were diluted 1∶5 with DNase free water and stored at −20°C prior to downstream applications.

### Genotyping of the UGT1A1 (TA)_n_ Repeat

The UGT1A1 (TA)_n_ repeat (rs8175347) was genotyped by pyrosequencing using a PyroMark Q24 (Qiagen, Venlo, The Netherlands). A 169-bp fragment of the promoter region of the UGT1A1 gene was amplified using a biotinylated forward primer 5′-AACAATAACTTGGTGTATCGATTGGT-3′ in combination with the reverse primer 5′-CAGCATGGGACACCACTG-3′ using PyroMark PCR Kit (cat. no. 978703, Qiagen). Each PCR reaction contained 1× PyroMark PCR Master Mix, 1×CoralLoad Concentrate, 1× Q-Solution, 4.0 mM MgCl_2_ and 0.2 µM of each primer in a total volume of 25 µl. The cycling conditions comprised 15 min polymerase activation at 95°C followed by 45 cycles of 30 seconds at 94°C, 58°C and 72°C and a final extension step at 72°C for 10 min. The PCR product (10 µl) was then used for pyrosequencing with a reverse sequencing primer 5′- TCGCCCTCTCCTACTTATAT-3′ on PyroMark Q24 according to the manufacturer's instructions. The samples were analyzed using PyroMark Q24 Software v2.0.6.

### Genotyping of Single Nucleotide Polymorphisms (SNPs)

To detect SNPs in the target nucleic acid sequences, allelic discrimination experiments using predesigned TaqMan® SNP Genotyping Assays (Life Technologies, Carlsbad, USA) were performed on a Viia7 Real-Time PCR System (Life Technologies). The allelic discrimination experiment includes three steps; a pre-PCR read at 60°C for 30 s to collect baseline fluorescence data, an amplification run (quantitative real-time PCR) and a post-PCR read at 60°C for 30 s to collect endpoint fluorescence data and determine the results for genotyping. The real-time PCR set-up was 12.5 µl 2×TaqMan Universal PCR Master Mix (cat. no. 4304437, Life Technologies), 1.25 µl TaqMan® SNP Genotyping Assay (See Table S2), 4 µl genomic DNA and 7.25 µl DNase free water. The PCR reaction comprised 10 min polymerase activation at 95°C followed by 40 cycles of 95°C for 15 s and 60°C for 1 min. A non-template control and positive controls, DNA samples with known genotype from Coriell Institute for Medical Research (See Table S3), were included in each run. After signal normalization and multicomponent analysis, the Viia7 RUO Software v1.2 graphed the results of the allelic discrimination runs on a scatterplot.

### Statistical Analysis

Power calculation: A sample size of 150 individuals with high bilirubin levels and 150 individuals with normal bilirubin level generates ≥80% power to detect a genotypic relative risk of ≥2 between the two bilirubin groups under an additive model (for allele frequencies of ≥0.1) (QUANTO, version 1.2).

Statistical analysis was performed using SPSS 16.0 statistic software (SPSS Inc., Chicago, USA). The Pearson chi-square test was used for testing categorical variables (SNP frequencies). The Benjamini-Hochberg false discovery rate (FDR) procedure was used for multiple testing correction (q<0.05) [19]. Univariate and multivariate forward conditional logistic regression analyses were performed to evaluate the impact of selected polymorphisms in UGT1A1, SLCO1B3, ABCC2 and NUP153 genes on bilirubin concentrations, after adjustments for covariates. The significance level of P≤0.05 was set for entry into the models and P≥0.10 for removal from the models.

For gene locus consisting of two or more SNPs (UGT1A1 and SLCO1B3), the pairwise correlation coefficients (r^2^) of linkage disequilibrium was calculated and plotted using JLIN (A Java based Linkage Disequilibrium plotter, version 1.6.0) [20]. Haplotypes and diplotypes for SNPs were estimated by PHASE (version 2.1) [21], [22]. Diplotypes were assigned to each individual with a probability cut off of 0.9 for assignment. Hardy-Weinberg equilibrium was tested for each individual SNP using either JLIN or Michael H. Court's (2005–2008) online calculator [23]. Two-sided exact Mann-Whitney tests were used to compare bilirubin values between two diplotype groups.

## Supporting Information

Figure S1 **Serum bilirubin concentrations in the NORIP population (n = 2884).**
(PPTX)

Table S1 **Cumulative distribution of UGT1A1-variants with increasing serum bilirubin concentration.**
(DOC)

Table S2 **TaqMan® SNP Genotyping Assays, Life Technologies.**
(XLSX)

Table S3 **Positive controls used in genotyping from Coriell Institute for Medical Research.**
(XLSX)
