# Supplementary material for: Serum Bilirubin Concentration in Healthy Adult North-Europeans Is Strictly Controlled by the UGT1A1 TA-Repeat Variants
Source: PLoS One. 2014 Feb 28;9(2):e90248. doi: 10.1371/journal.pone.0090248 (PMC3938665; doi:10.1371/journal.pone.0090248)
Supplement: Table S1 — Cumulative distribution of UGT1A1-variants with increasing serum bilirubin concentration. (DOC) [file pone.0090248.s002.doc]

Table S1.

Cumulative distribution of UGT1A1-variants with increasing serum bilirubin concentration*

| **Bilirubin** (μmol/L) | Percentile | **Cumulative distribution**  **(%)** | | |
| --- | --- | --- | --- | --- |
|  |  | 6/6 | 6/7 | 7/7 |
| 7.4 | 26.0 | 18.6 | 7.4 | 0.0 |
| 9.8 | 53.2 | 28.4 | 24.7 | 0.0 |
| 10.1 | 56.3 | 29.7 | 26.6 | 0.0 |
| 10.9 | 60.6 | 30.9 | 29.7 | 0.0 |
| 11.6 | 65.5 | 32.8 | 32.2 | 0.6 |
| 12.1 | 69.9 | 33.4 | 35.2 | 1.2 |
| 13.0 | 74.8 | 34.6 | 38.3 | 1.9 |
| 14.4 | 79.8 | 36.5 | 41.4 | 1.9 |
| 14.9 | 85.9 | 38.3 | 45.1 | 2.5 |
| 17.3 | 90.3 | 39.6 | 48.2 | 2.5 |
| 17.4 | 91.5 | 39.6 | 48.8 | 3.1 |
| 18.3 | 92.0 | 39.6 | 49.0 | 3.4 |
| 19.2 | 93.1 | 39.6 | 49.4 | 4.1 |
| 20.6 | 94.0 | 39.7 | 49.8 | 4.5 |
| 22.1 | 95.0 | 39.7 | 50.1 | 5.2 |
| 24.1 | 96.0 | 39.7 | 50.5 | 5.7 |
| 25.9 | 97.1 | 39.8 | 50.6 | 6.7 |
| 29.4 | 98.1 | 39.8 | 50.7 | 7.5 |
| 33.0 | 99.0 | 39.8 | 50.7 | 8.4 |
| 59.9 | 100.0 | 39.8 | 50.9 | 9.3 |

*Of the NORIP population: Estimated from the data of Fig 1. The normal and high bilirubin groups represent the lower 91.5 and upper 8.5 percent of this population.
